# CRISPR-based screening identifies *XPO7* as a positive regulator of senescence

**DOI:** 10.1093/procel/pwad012

**Published:** 2023-03-10

**Authors:** Lan-Zhu Li, Kuan Yang, Yaobin Jing, Yanling Fan, Xiaoyu Jiang, Si Wang, Guang-Hui Liu, Jing Qu, Shuai Ma, Weiqi Zhang

**Affiliations:** State Key Laboratory of Membrane Biology, Institute of Zoology, Chinese Academy of Sciences, Beijing 100101, China; University of Chinese Academy of Sciences, Beijing 100049, China; Institute for Stem Cell and Regeneration, Chinese Academy of Sciences, Beijing 100101, China; Beijing Institute for Stem Cell and Regenerative Medicine, Beijing 100101, China; CAS Key Laboratory of Genomic and Precision Medicine, Beijing Institute of Genomics, Chinese Academy of Sciences and China National Center for Bioinformation, Beijing 100101, China; University of Chinese Academy of Sciences, Beijing 100049, China; Sino-Danish College, University of Chinese Academy of Sciences, Beijing 101408, China; State Key Laboratory of Membrane Biology, Institute of Zoology, Chinese Academy of Sciences, Beijing 100101, China; University of Chinese Academy of Sciences, Beijing 100049, China; Institute for Stem Cell and Regeneration, Chinese Academy of Sciences, Beijing 100101, China; Beijing Institute for Stem Cell and Regenerative Medicine, Beijing 100101, China; School of Future Technology, University of Chinese Academy of Sciences, Beijing 100190, China; CAS Key Laboratory of Genomic and Precision Medicine, Beijing Institute of Genomics, Chinese Academy of Sciences and China National Center for Bioinformation, Beijing 100101, China; University of Chinese Academy of Sciences, Beijing 100049, China; State Key Laboratory of Membrane Biology, Institute of Zoology, Chinese Academy of Sciences, Beijing 100101, China; University of Chinese Academy of Sciences, Beijing 100049, China; Institute for Stem Cell and Regeneration, Chinese Academy of Sciences, Beijing 100101, China; Beijing Institute for Stem Cell and Regenerative Medicine, Beijing 100101, China; Advanced Innovation Center for Human Brain Protection and National Clinical Research Center for Geriatric Disorders, Xuanwu Hospital Capital Medical University, Beijing 100053, China; Aging Translational Medicine Center, International Center for Aging and Cancer, Beijing Municipal Geriatric Medical Research Center, Xuanwu Hospital, Capital Medical University, Beijing 100053, China; State Key Laboratory of Membrane Biology, Institute of Zoology, Chinese Academy of Sciences, Beijing 100101, China; University of Chinese Academy of Sciences, Beijing 100049, China; Institute for Stem Cell and Regeneration, Chinese Academy of Sciences, Beijing 100101, China; Beijing Institute for Stem Cell and Regenerative Medicine, Beijing 100101, China; School of Future Technology, University of Chinese Academy of Sciences, Beijing 100190, China; Advanced Innovation Center for Human Brain Protection and National Clinical Research Center for Geriatric Disorders, Xuanwu Hospital Capital Medical University, Beijing 100053, China; Aging Translational Medicine Center, International Center for Aging and Cancer, Beijing Municipal Geriatric Medical Research Center, Xuanwu Hospital, Capital Medical University, Beijing 100053, China; State Key Laboratory of Stem Cell and Reproductive Biology, Institute of Zoology, Chinese Academy of Sciences, Beijing 100101, China; University of Chinese Academy of Sciences, Beijing 100049, China; Institute for Stem Cell and Regeneration, Chinese Academy of Sciences, Beijing 100101, China; Beijing Institute for Stem Cell and Regenerative Medicine, Beijing 100101, China; State Key Laboratory of Membrane Biology, Institute of Zoology, Chinese Academy of Sciences, Beijing 100101, China; University of Chinese Academy of Sciences, Beijing 100049, China; Institute for Stem Cell and Regeneration, Chinese Academy of Sciences, Beijing 100101, China; Beijing Institute for Stem Cell and Regenerative Medicine, Beijing 100101, China; CAS Key Laboratory of Genomic and Precision Medicine, Beijing Institute of Genomics, Chinese Academy of Sciences and China National Center for Bioinformation, Beijing 100101, China; University of Chinese Academy of Sciences, Beijing 100049, China; Institute for Stem Cell and Regeneration, Chinese Academy of Sciences, Beijing 100101, China; School of Future Technology, University of Chinese Academy of Sciences, Beijing 100190, China; Sino-Danish College, University of Chinese Academy of Sciences, Beijing 101408, China

**Dear Editor,**

Cells enter senescence, or irreversible growth arrest, when exposed to stressors such as DNA damage, epigenetic alterations and chronic inflammation (Zhao and Chen, 2022). In aging and aging-related diseases, senescent cells are known to accumulate across tissues and organs (Sun et al., 2022; Lopez-Otin et al., 2023). Although stem cells to a certain extent are capable of repairing and regenerating aged or injured tissues through their self-renewal and differentiation abilities, with aging, their function declines, which contributes to aging phenotypes in many tissues (Cai et al., 2022b). To understand the mechanisms underlying senescence, genetic and epigenetic changes have been investigated in aging models (Zhang et al., 2020; Cai et al., 2022a). Such efforts are crucial for identifying key regulators that can be targeted as intervention therapies in stem cell senescence and aging-related disorders.

The transport of macromolecules between the cell’s nucleus and cytoplasm is a regulated mechanism essential for eukaryotic organisms. It requires a sophisticated barrier system with channels (nuclear pore complexes, NPCs) embedded in the nuclear envelope, and shuttling nuclear transport receptors (NTRs) that transfer cargoes. The NPCs consist of a cytoplasmic ring, the inner pore ring, the nuclear ring and peripheral elements (nuclear basket and cytoplasmic filaments) (Yang et al., 2014). NTRs contain import proteins (importins) and export proteins (exportins) that control cargo transport into or out of the nucleus. Importantly, the nuclear transport system also participates in nuclear and cytoplasmic processes, such as chromatin organization, epigenetic regulation, transcription, mRNA maturation, spliceosome and ribosome assembly. These diverse roles make the nuclear transport system a hot spot for disruptions that is linked with diseases ranging from neurodegenerative and cardiovascular disorders to autoimmune dysfunctions and aggressive cancers (Dickmanns et al., 2015). However, to date, our understanding of relationships between the functions of nuclear transport system and stem cell senescence is limited. Here, using CRISPR-based screening for nuclear transport system-associated genes (NTSAGs), we identified that deficiency of the transport protein XPO7 (Exportin 7, also termed RANBP16) alleviates human mesenchymal stem cell (hMSC) senescence. We also demonstrated that XPO7 functions as a destabilizer for HDAC2 (histone deacetylase 2), thereby contributing to cellular senescence.

To systematically define components of the nucleocytoplasmic transport system that affect by human MSC aging, we constructed a CRISPR-based loss-of-function (LOF) screening library (NTSAG library), which contained 208 sgRNAs targeting 32 nucleoporins and 34 NTRs with three sgRNAs per gene and 10 non-targeting controls (sg-*NTCs*) (Figs. 1A and S1A). Then, we applied CRISPR screening in three types of senescent stem cell models: replicative senescent hMSCs (RS hMSCs), WS (Werner syndrome, *WRN*-deficient) and HGPS (Hutchinson-Gilford progeria syndrome, bearing the heterozygous *LMNA*^*G608G*/+^ mutation) hMSCs; the latter two of which are human stem cell models of premature aging (Wu et al., 2018). In the intermediate passage of RS hMSCs, WS hMSCs, and HGPS hMSCs, we infected the NTSAG lentiviral library at a low multiplicity of infection (MOI ≈ 0.3) to ensure that one cell was infected with at most one sgRNA. At the same time, equivalent cells were infected with sg-*NTC* lentivirus at the same MOI as the control. After selection with puromycin, we serially passaged cells until controls exhibited growth arrest and harvested these cells. We then constructed DNA libraries to discover enriched genes whose knockdown prevented hMSC senescence. Coupled with three distinct screenings, we identified *XPO7*, a broad-spectrum bidirectional transporter (Aksu et al., 2018), as the only hit enriched in RS-, WS- and HGPS-based models (Figs. 1B–E, S1B and S1C; Table S1). To confirm that XPO7 deficiency is inversely correlated with aging, we utilized lentivirus-mediated CRISPR knockout (CRISPRko) to diminish XPO7 expression in RS, WS and HGPS hMSCs, respectively (Fig. 1F). Consistently, the depletion of *XPO7* ameliorated the onset of growth arrest, as evidenced by a higher percentage of Ki67-positive cells and increased clonal expansion ability in all three kinds of senescent hMSCs (Fig. 1G, 1H, 1K, 1L, 1O and 1P). Moreover, XPO7 depletion alleviated multiple senescent phenotypes, as manifested by decreased numbers of senescence-associated β-galactosidase (SA-β-gal)-positive cells, reduced expression of the aging marker p21 and increased expression of nuclear Lamin B1, LAP2 and H3K9me3 as well as reduced secretion of senescence-associated secretory phenotype (SASP) associated protein (IL-6, interleukin-6) (Figs. 1I, 1J, 1M, 1N, 1Q, 1R and S1D–I). Thus, our results suggest an important role for XPO7 in stem cell senescence.

**Figure 1.**
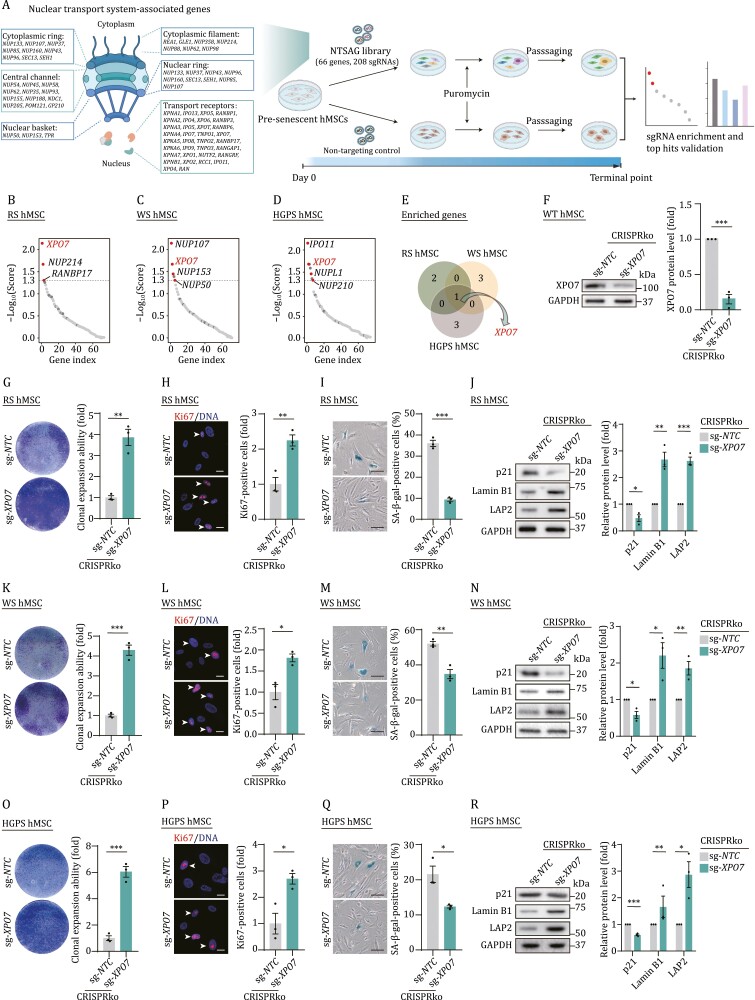
**CRISPR-based screening identifies XPO7 as a driver of cellular senescence**. (A) Schematic of CRISPR-based screening to identify pro-senescence genes in RS, WS and HGPS hMSCs. (B) Scatter plot showing the enriched genes in three replicate screens in RS hMSCs. The red dots indicate the significantly enriched genes, the dark gray dots indicate non-targeting control sgRNAs. (C) Scatter plot showing the enriched genes in three replicate screens in WS hMSCs. The red dots indicate the significantly enriched genes, the dark gray dots indicate non-targeting control sgRNAs. (D) Scatter plot showing the enriched genes in three replicate screens in HGPS hMSCs. The red dots indicate the significantly enriched genes, the dark gray dots indicate non-targeting control sgRNAs. (E) Venn diagram showing the number of genes enriched in CRISPR-based screening in RS-, WS- and HGPS-hMSCs. (F) Western blot analysis of XPO7 protein level in WT hMSCs after CRISPR-mediated knockout (CRISPRko) of XPO7. Data are presented as the mean ± SEM. *n =* 3 independent experiments. ***P < 0.001. (G) Clonal expansion analysis of RS hMSCs after CRISPR-mediated knockout (CRISPRko) of XPO7. Data are presented as the mean ± SEM. *n =* 3 biological replicates. **P < 0.01. (H) Immunofluorescence analysis of Ki67 in RS hMSCs after CRISPR-mediated knockout (CRISPRko) of XPO7. Scale bars, 20 μm. White arrows indicate Ki67-positive cells. Data are presented as the mean ± SEM. *n =* 3 biological replicates. **P < 0.01. (I) SA-β-gal staining of RS hMSCs after CRISPR-mediated knockout (CRISPRko) of XPO7. Scale bars, 100 μm. Data are presented as the mean ± SEM. *n =* 3 biological replicates. ***P < 0.001. (J) Western blot analysis of the indicated protein levels in RS hMSCs after CRISPR-mediated knockout (CRISPRko) of XPO7. Data are presented as the mean ± SEM. *n =* 3 independent experiments. *P < 0.05; **P < 0.01; ***P < 0.001. (K) Clonal expansion analysis of WS hMSCs after CRISPR-mediated knockout (CRISPRko) of XPO7. Data are presented as the mean ± SEM. *n =* 3 biological replicates. ***P < 0.001. (L) Immunofluorescence analysis of Ki67 in WS hMSCs after CRISPR-mediated knockout (CRISPRko) of XPO7. Scale bars, 20 μm. White arrows indicate Ki67-positive cells. Data are presented as the mean ± SEM. *n =* 3 biological replicates. *P < 0.05. (M) SA-β-gal staining of WS hMSCs after CRISPR-mediated knockout (CRISPRko) of XPO7. Scale bars, 100 μm. Data are presented as the mean ± SEM. *n =* 3 biological replicates. **P < 0.01. (N) Western blot analysis of the indicated protein levels in WS hMSCs after CRISPR-mediated knockout (CRISPRko) of XPO7. Data are presented as the mean ± SEM. *n =* 3 independent experiments. *P < 0.05; **P < 0.01. (O) Clonal expansion analysis of HGPS hMSCs after CRISPR-mediated knockout (CRISPRko) of XPO7. Data are presented as the mean ± SEM. *n =* 3 biological replicates. ***P < 0.001. (P) Immunofluorescence analysis of Ki67 in HGPS hMSCs after CRISPR-mediated knockout (CRISPRko) of XPO7. Scale bars, 20 μm. White arrows indicate Ki67-positive cells. Data are presented as the mean ± SEM. *n =* 3 biological replicates. *P < 0.05. (Q) SA-β-gal staining of HGPS hMSCs after CRISPR-mediated knockout (CRISPRko) of XPO7. Scale bars, 100 μm. Data are presented as the mean ± SEM. *n =* 3 biological replicates. *P < 0.05. (R) Western blot analysis of the indicated protein levels in HGPS hMSCs after CRISPR-mediated knockout (CRISPRko) of XPO7. Data are presented as the mean ± SEM. *n =* 3 independent experiments. *P < 0.05; **P < 0.01; ***P < 0.001.

To investigate the role of XPO7 in regulating human stem cell senescence, we next generated XPO7-deficient human embryonic stem cells (*XPO7*^−/−^ hESCs) by CRISPR/Cas9-based gene editing (Fig. S2A). As validated by western blot analysis, the gene targeting at the XPO7 locus was successful (Fig. S2B). XPO7 deficiency did not affect cellular proliferative ability in hESCs (Fig. S2C). Furthermore, *XPO7*^−/−^ hESCs expressed typical pluripotency markers, including NANOG, OCT4 and SOX2, at levels comparable to wild type hESCs (WT, *XPO7*^+/+^) (Fig. S2D). In addition, karyotype and genome-wide copy number variation (CNV) analyses showed that the genomic integrity was maintained in *XPO7*^−/−^ hESCs (Fig. S2E and S2F). Altogether, these indicate that XPO7 is dispensable for maintaining hESC homeostasis.

Next, we differentiated *XPO7*^+/+^ and *XPO7*^−/−^ hESCs into hMSCs (Fig. S2G). Both *XPO7*^+/+^ and *XPO7*^−/−^ hMSCs were positive for classic hMSC surface markers including CD73, CD90 and CD105 (Fig. S2H). The lack of XPO7 protein was confirmed by western blot analysis (Fig. 2A). Similar to *XPO7*^+/+^ hMSCs, *XPO7*^−/−^ hMSCs were able to differentiate into osteoblasts, adipocytes and chondrocytes, although with a differentiation bias towards the osteoblast fate (Fig. S2J–L). The genomic integrity was also maintained in *XPO7*^−/−^ hMSCs (Fig. S2I). Similarly, senescent phenotypes were attenuated in *XPO7*^−/−^ hMSCs (Figs. 2B–I and S2M) as observed in hMSCs with lentivirus-mediated XPO7 deficiency. Consistent with these phenotypic reversals of senescence, we observed differentially expressed genes (DEGs) upon XPO7 deficiency (Table S1). Genes associated with cell proliferation and cell division were upregulated while inflammatory and SASP-related genes were downregulated (Figs. 2J and S2N–P). Moreover, in other senescent models induced by ultraviolet (UV) irradiation, H_2_O_2_ and oncogene activation (Fig. S3A, S3E and S3I), XPO7 deficiency could alleviate senescent phenotypes as determined by decreased SA-β-gal positive cells (Fig. S3B, S3F and S3J), increased Ki67-positive cells (Fig. S3C, S3G and S3K) and clonal expansion (Fig. S3D, S3H and S3L). Whereas the ablation of *XPO7* retarded senescence of human dermal fibroblasts (Fig. S4A–D). Taken together, these observations indicate that *XPO7* is a key regulator that promotes senescence in diverse biological contexts.

**Figure 2.**
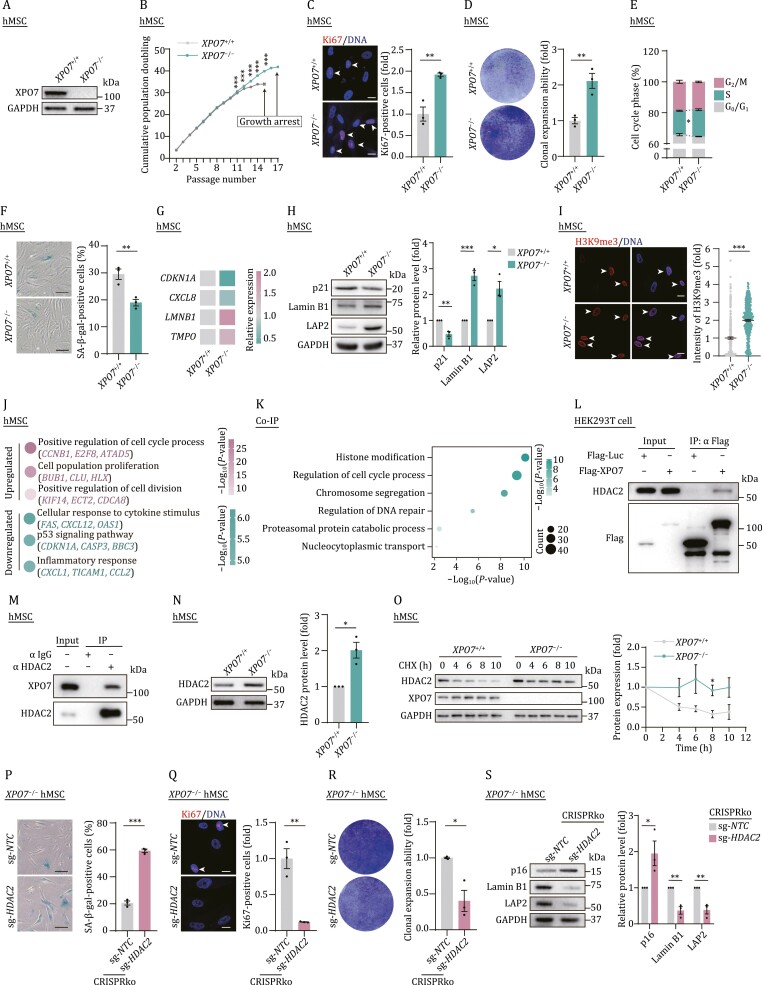
**XPO7 regulates hMSC senescence by affecting HDAC2 protein stability**. (A) Western blot analysis of XPO7 in *XPO7*^+/+^ and *XPO7*^−/−^ hMSCs. (B) Growth curve analysis of *XPO7*^+/+^ and *XPO7*^−/−^ hMSCs. Data are presented as the mean ± SEM. *n =* 3 biological replicates. ***P < 0.001. (C) Immunofluorescence analysis of Ki67 in *XPO7*^+/+^ and *XPO7*^−/−^ hMSCs. Scale bars, 20 μm. White arrows indicate Ki67-positive cells. Data are presented as the means ± SEM. *n =* 3 biological replicates. **P < 0.01. (D) Clonal expansion analysis of *XPO7*^+/+^ and *XPO7*^−/−^ hMSCs. Data are presented as the means ± SEM. *n =* 3 biological replicates. **P < 0.01. (E) Cell cycle analysis of *XPO7*^+/+^ and *XPO7*^−/−^ hMSCs. Data are presented as the means ± SEM. *n =* 3 biological replicates. *P < 0.05. (F) SA-β-gal staining of *XPO7*^+/+^ and *XPO7*^−/−^ hMSCs. Scale bars, 100 μm. Data are presented as the mean ± SEM. *n =* 3 biological replicates. **P < 0.01. (G) Heatmap showing the RT-qPCR detection of the relative mRNA levels for the indicated genes in *XPO7*^+/+^ and *XPO7*^−/−^ hMSCs. (H) Western blot analysis of the indicated protein levels in *XPO7*^+/+^ and *XPO7*^−/−^ hMSCs. Data are presented as the mean ± SEM. *n =* 3 independent experiments. *P < 0.05; **P < 0.01; ***P < 0.001. (I) Immunofluorescence analysis of H3K9me3 in *XPO7*^+/+^ and *XPO7*^−/−^ hMSCs. Scale bars, 20 μm. White arrowheads denote the cells with increased H3K9me3 signals. Data are presented as the mean ± SEM. *n* = 300 cells from three biological replicates. ***P < 0.001. (J) Point plot showing GeneOntology (GO) terms and pathways enriched by upregulated (pink) and downregulated (green) DEGs in *XPO7*^−/−^ hMSCs. (K) GO enrichment analysis of XPO7-interacting proteins identified by mass spectrometry. (L) Co-IP analysis to verify the interaction between HDAC2 and FLAG-XPO7 in HEK293T cells. (M) Co-IP analysis of the interaction between HDAC2 and XPO7 in hMSCs. (N) Western blot analysis of HDAC2 in *XPO7*^+/+^ and *XPO7*^−/−^ hMSCs. Data are presented as the mean ± SEM. *n =* 3 independent experiments. *P < 0.05. (O) Protein stability analysis of HDAC2 in *XPO7*^+/+^ and *XPO7*^−/−^ hMSCs. Protein levels of HDAC2 at indicated time points after treatment with a protein synthesis inhibitor cycloheximide (CHX) were determined by western blot. Data are presented as the mean ± SEM. *n =* 3 independent experiments. *P < 0.05. (P) SA-β-gal staining of *XPO7*^−/−^ hMSCs after CRISPR-mediated knockout (CRISPRko) of HDAC2. Scale bars, 100 μm. Data are presented as the mean ± SEM. *n =* 3 biological replicates. ***P < 0.001. (Q) Immunofluorescence analysis of Ki67 in *XPO7*^−/−^ hMSCs after CRISPR-mediated knockout (CRISPRko) of HDAC2. Scale bars, 20 μm. White arrows indicate Ki67-positive cells. Data are presented as the mean ± SEM. *n =* 3 biological replicates. **P < 0.01. (R) Clonal expansion analysis of *XPO7*^−/−^ hMSCs after CRISPR-mediated knockout (CRISPRko) of HDAC2. Data are presented as the mean ± SEM. *n =* 3 biological replicates. *P < 0.05. (S) Western blot analysis of the indicated protein levels in *XPO7*^−/−^ hMSCs after CRISPR-mediated knockout (CRISPRko) of HDAC2. Data are presented as the mean ± SEM. *n =* 3 independent experiments. *P < 0.05; **P < 0.01.

To evaluate the effect of XPO7 activation on hMSC senescence, we used CRISPR-mediated activation (CRISPRa) to elevate XPO7 expression in early-passage WT hMSCs (young hMSCs) (Fig. S5A and S5B). As expected, the activation of XPO7 led to senescent phenotypes, such as decreased cell proliferation and increased SA-β-gal activity (Fig. S5C–E). Consistent with the accelerated senescence characteristics, XPO7 activation induced expression of *CDKN1A* and SASP-related genes (e.g., *IL6* and *CXCL8*) and increased IL-6 secretion (Fig. S5F–H). Additionally, elevated XPO7 expression was associated with diminished expression of nuclear lamina-associated genes (*LMNB1*, *TMPO*) and with reduced H3K9me3 (Fig. S5F, S5G and S5I). Moreover, ectopic expression of XPO7 with the cDNA transgene phenocopied the accelerated senescence observed in hMSCs with XPO7 activation (Fig. S5J–N). Furthermore, activation of XPO7 expression in human dermal fibroblasts could also enhance the senescent phenotypes, such as decreased cell proliferation, increased SA-β-gal activity and reduced H3K9me3 (Fig. S4E–H). Collectively, these results indicate XPO7 as a potential driver of human stem cell senescence.

To investigate the molecular mechanism by which XPO7 deficiency attenuated hMSC senescence, we sought to identify XPO7-interacting proteins. Thus, we ectopically expressed FLAG-tagged XPO7 protein in HEK293T cells and performed Co-immunoprecipitation (Co-IP) with FLAG antibody followed by mass spectrometry analysis (IP-MS). Within our dataset of XPO7-interacting proteins, we observed a subset that was associated with chromosome organization and histone modification (Fig. 2K; Table S2). Among these proteins, we focused on HDAC2, a histone deacetylase that is downregulated with aging (Warnon et al., 2021) (Fig. S6A). The interaction between XPO7 and HDAC2 was validated through exogenous and endogenous Co-IP assays (Fig. 2L and 2M). Then we asked whether HDAC2 protein level might be influenced by XPO7 depletion. Indeed, we observed that the expression of HDAC2 was increased upon XPO7 depletion and decreased upon XPO7 activation (Figs. 2N, S6D, S6F, S6H and S6I). However, we did not detect a difference of *HDAC2* mRNA level in XPO7-deficient hMSCs as revealed by RT-qPCR (Fig. S6B, S6C, S6E and S6G). Thus, we hypothesized that XPO7 might regulate hMSC senescence by destabilizing the HDAC2 protein. Indeed, when we treated *XPO7*^−/−^ hMSCs with cycloheximide (CHX, a protein synthesis inhibitor), we observed that XPO7 deficiency led to increased protein stability of HDAC2 (Fig. 2O). To confirm the role of XPO7 in regulating HDAC2 stability, we monitored HDAC2 turnover in XPO7-overexpressed hMSCs. The results showed that XPO7 triggered the degradation of HDAC2 (Fig. S6J), suggesting that a functional link exists between XPO7 and HDAC2 during hMSC senescence. Next, we explored whether the decline of protein stability of HDAC2 is in a XPO7-mediated manner. To this end, we treated XPO7-overexpressed hMSCs with the proteasome inhibitor MG132, and found overexpression of XPO7 reduced HDAC2 protein level, which was abolished by MG132 (Fig. S6K). Together these results indicate that XPO7 facilitates proteasome-dependent HDAC2 stabilization.

To further determine the role of HDAC2 upon hMSC senescence, we reduced HDAC2 levels using CRISPRko in young hMSCs (Fig. S6L). As evidenced by impaired proliferation, increased SA-β-gal activity and downregulation of senescent markers Lamin B1 and LAP2, and increased expression of p16, loss of HDAC2 accelerated hMSC senescence (Fig. S6M–P), similar to the effects of XPO7 overexpression. Consistently, depletion of HDAC2 typically caused senescent phenotypes in *XPO7*^−/−^ hMSCs, such as decreased proliferative ability (compromised clonal expansion and reduced proportion of Ki67-positive cells), increased SA-β-gal positive cells, repressed expression of Lamin B1 and LAP2 and increased the expression of p16 (Fig. 2P–S). Collectively, these data suggest that HDAC2 is a critical downstream XPO7 mediator that reinforces cellular senescence.

To our knowledge, this is the first study that identifies nuclear transport system-related genes involved in stem cell senescence using CRISPR/Cas9-based LOF screening. Our results revealed that ablation of the transport receptor XPO7 ameliorates hMSC senescence. The nuclear transport system facilitates import of transcription factors, core histones and export of ribosomes that are required for gene regulation, DNA replication and translation, respectively (Dickmanns et al., 2015). Given that the nuclear transport system is critically involved in cellular hemostasis and its defective function associated with human diseases, a gain-of-function screen might help uncover novel insights into the functions of nuclear transport system during aging.

XPO7 controls proteins shuttling between the nucleus and cytoplasm, but little is known about the function of XPO7 in stem cell senescence. Recently, it was described that XPO7 affects TCF3 protein level to induce p21 during oncogene-induced senescence (OIS) (Innes et al., 2021), while the mechanisms by which XPO7 regulates TCF3 remains unclear. Moreover, previous studies reported that XPO7 transfers core histones from the nucleus to cytoplasm (Hattangadi et al., 2014), but the functions of XPO7 upon epigenetic modification are still unknown. Our study presents the first evidence that XPO7 impairs HDAC2 proteostasis and thereby accelerates cellular senescence in human MSCs. In support of our findings, HDAC2 was described to protect against cellular senescence by regulating the expression of prosenescent genes (e.g., *p16* and *p21*), DNA damage response and SASP related genes via epigenetic mechanisms (Zhou et al., 2008). Post-translational modification is closely related to HDAC2 protein level and activity, such as phosphorylation and nitration (Colussi et al., 2008). Further study is required to investigate whether the posttranslational modification is involved in XPO7-mediated HDAC2 degradation. Traditionally, XPO7 works as a transport receptor, thus deletion of XPO7 might also affect its transport capability. Therefore, exploring the homeostasis of cargoes with XPO7 deficiency might provide new clues to mechanisms against hMSC senescence.

Collectively, we performed a CRISPR/Cas9-based LOF screening for nuclear transport system-related genes in regulating human MSC senescence and identified XPO7 as a new mediator of hMSC senescence. Our analyses also provide a potential intervention target for aging-related diseases using new therapeutic strategies, such as gene delivery and small molecular inhibitors.

## Supplementary Material

pwad012_suppl_Supplementary_Materials

pwad012_suppl_Supplementary_Table_S1

pwad012_suppl_Supplementary_Table_S2

pwad012_suppl_Supplementary_Table_S3

## Footnotes

We are grateful to Lei Bai, Ruijun Bai, Qun Chu, Xiangmei Jin, Luyang Tian, Jing Lu and Ying Yang for their administrative assistance. We thank Jifeng Wang (IBP, CAS), for the help in LC-MS/MS assays, Junying Jia (IBP, CAS), Shu Meng (IBP, CAS) and Qing Meng (IOZ, CAS) for their assistance in flow cytometry analyses and Wei Wang for providing WS hMSCs. We also thank the Biorender platform (biorender.com) which was used for schematic diagram.

This work was supported by the National Key Research and Development Program of China (2020YFA0804000), the Strategic Priority Research Program of the Chinese Academy of Sciences (XDA16000000), the National Natural Science Foundation of China (81921006, 82125011, 92149301, 92168201, 91949209, 92049304, 92049116, 32121001, 82271600, 82192863, 32000500, 82122024, 82071588, 32000510), the National Key Research and Development Program of China (2018YFC2000100, 2018YFA0107203, 2020YFA0112200, 2021YFF1201005, the STI2030-Major Projects-2021ZD0202400, 2022YFA1103700), CAS Project for Young Scientists in Basic Research (YSBR-076, YSBR-012), the Program of the Beijing Natural Science Foundation (Z190019), K. C. Wong Education Foundation (GJTD-2019-06, GJTD-2019-08), The Pilot Project for Public Welfare Development and Reform of Beijing-affiliated Medical Research Institutes (11000022T000000461062), Youth Innovation Promotion Association of CAS (E1CAZW0401, 2022083), Young Elite Scientists Sponsorship Program by CAST (YESS20200012, YESS20210002), the Informatization Plan of Chinese Academy of Sciences (CAS-WX2021SF-0301, CAS-WX2022SDC-XK14, CAS-WX2021SF-0101), and the Tencent Foundation (2021-1045).

The authors declare no competing interests. All institutional and national guidelines for the care were followed. The authors declare their agreement to participate and agreement to publish.

All data associated with this study are present in the paper or the Supplementary Materials. The raw data of screening, WGS data and transcriptomic data obtained have been deposited in the Genome Sequence Archive in the National Genomics Data Center, Beijing Institute of Genomics (China National Center for Bioinformation) of the Chinese Academy of Sciences, under accession number HRA003304 and HRA003369. The raw data of LC-MS/MS data have been deposited to the ProteomeXchange Consortium via the iProX partner repository under accession number PXD037890.

No code or model was generated in this study.

G.-H.L., J.Q., S.M. and W.Z. conceptualized the work and supervised overall experiments. L.-Z.L. performed the CRISPR screening, phenotypic and mechanistic analyses. K.Y. and X.J. performed bioinformatics analyses. Y.J. performed plasmid construction, RT-qPCR and Co-IP/MS sample preparation. Y.F. and S.W. performed immunofluorescence staining, cell culture and differentiation and primary fibroblast isolation. G-H.L., J.Q., S.M., W.Z., L.-Z.L. and Y.F. performed data analysis. G.-H.L., J.Q., S.M., W.Z., L.-Z.L., Y.J. and K.Y. performed manuscript writing, review, and editing. All authors reviewed the manuscript.
