# Supplementary material for: CRISPR-based screening identifies XPO7 as a positive regulator of senescence
Source: Protein Cell. 2023 Mar 10;14(8):623–8. doi: 10.1093/procel/pwad012 (PMC10392025; doi:10.1093/procel/pwad012)
Supplement: pwad012_suppl_Supplementary_Materials [file pwad012_suppl_supplementary_materials.pdf]

Supplemental Materials

Materials and methods

Cell culture

WT hESCs (line H9, WiCell Research) and *XPO7*$^{-/-}$ hESCs were maintained on mitomycin C-inactivated mouse embryonic fibroblasts (MEFs) with ESC culture medium (80% DMEM/F12 (Gibco), 20% Knockout Serum Replacement (Gibco), 1% non-essential amino acids (NEAA, Gibco), 1% GlutaMAX (Gibco), 1% penicillin/streptomycin (Gibco), 55 μmol/L β-mercaptoethanol (Invitrogen), and 10 ng/mL fibroblast growth factor 2 (FGF2, Joint Protein Central)); hESCs were also cultured on plates coated with Matrigel (BD Biosciences) in the mTeSR medium (STEMCELL Technologies, Vancouver).

hMSCs were cultured on plates coated with 0.1% gelatin in MSC medium (90% α-MEM with GlutaMAX (Gibco), 10% fetal bovine serum (FBS, Gibco), 1% NEAA, 1% penicillin/streptomycin and 1 ng/mL FGF2). WT hMSCs at late passage (LP, P > 12) are defined as replicative senescent (RS), and prematurely senescent HGPS and WS hMSCs at P 8-9 exhibit growth arrest as previous reports (Wu et al., 2018; Zhang et al., 2015).

HEK293T cells were cultured in DMEM/High Glucose (HyClone) with 10% FBS and 1% penicillin/streptomycin.

Human primary fibroblasts were isolated from human skin as previously described (Zou et al., 2021). Firstly, fresh skin samples were washed with PBS and incubated in 5 mg/mL dispase (Gibco) overnight. After that, dermis was digested by collagenase IV (Gibco). Then, fibroblasts were harvested and washed by PBS. Human primary fibroblasts were cultured in DMEM/High Glucose (HyClone) with 10% FBS, 1% penicillin/streptomycin and 1% NEAA. Human primary fibroblasts are replicative senescent in their late passage (P > 12).

Generation of *XPO7*$^{-/-}$ hESCs by CRISPR/Cas9-based strategy

XPO7$^{-/-}$ hESCs were generated by CRISPR/Cas9-based gene editing as previously described (Zhang et al., 2022). Briefly, sgRNA targeting the second exon of *XPO7* gene was cloned into the pCAG-mCherry-sgRNA vector (Addgene # 87110). Then, the reconstructed pCAG-mCherry-sgRNA vector and the pCAG-1BPNLS-Cas9-1BPNLS-2AGFP vector (Addgene # 87109) were electroporated into *XPO7*$^{+/+}$ hESCs by 4D-Nucleofector (Lonza). After that, hESCs were seeded on matrigel-coated plates and cultured with mTeSR medium supplemented containing ROCK inhibitor Y-27632 (Tocris) for 48 hr. mCherry/GFP-double-positive cells were sorted by flow cytometer (BD, Aria II) and cultured on mitomycin C-inactivated MEFs with ESC culture medium.

Emerging hESC clones were processed for genomic DNA extraction, PCR amplification and DNA sequencing. sgRNA sequences for gene editing and primers for clone identification are listed in Table S3.

Generation of hMSCs by directed differentiation from hESCs

XPO7$^{+/+}$, *XPO7*$^{-/-}$, WS and HGPS hMSCs were differentiated from corresponding hESCs as previously described (Liang et al., 2022). hESCs were dissociated into embryoid bodies (EBs), after which EBs were treated with MSC differentiation medium (90% MEMα, 10% FBS, 10 ng/mL FGF2, and 5 ng/mL TGFβ (HumanZyme) until fibroblast-like cells appeared. After that, cells were purified by FACS with antibodies corresponding to hMSC markers (CD73, CD90 and CD105). Antibodies used for flow cytometry analysis include anti-CD73-PE (BD Biosciences, 550257), anti-CD90-FITC (BD Biosciences, 555595), and anti-CD105-APC (BioLegend, 800508).

NTSAG library construction and plasmid construction

The NTSAG library targets 66 genes including nucleoporins and nuclear transport receptors. Each gene was targeted by three sgRNAs. The whole library contains 208 sgRNAs with 10 non-targeting controls. The sgRNAs were cloned into the lentiCRISPRv2 transfer plasmid (Addgene # 52961). After that, the plasmids were pooled together with equal mass. The sequences of sgRNAs are provided in Table S3.

To generate the lentiviral vectors expressing sgRNA targeting *XPO7* or *HDAC2*, non-targeting control sgRNA (sg-*NTC*) or specific sgRNA were cloned into the lentiCRISPRv2 transfer plasmid according to the manufacturer's instructions. To activate endogenous XPO7, sgRNA targeting *XPO7* was cloned into lentiSAMv2 vector (Addgene # 75112). To generate the XPO7 expression plasmids, cDNAs from *XPO7*$^{+/+}$ hMSCs were used as the template to amplify XPO7-Flag cDNA. After that, the XPO7-Flag cDNA was cloned into the pLE4 empty vector (a kind gift from Dr. Tomoaki Hishida). The primers used for cloning are listed in Table S3.

CRISPR/Cas9-based NTSAG library screen

The CRISPR/Cas9-based library screen was performed as described previously (Wang et al., 2021). Firstly, HEK293T cells were transfected with the sgRNA transfer plasmid and lentiviral packaging plasmids, pMD2.G (Addgene # 12259) and psPAX2 (Addgene # 12260) using Lipofectamine 3000 transfection reagent. Then the viral vectors were concentrated and titrated. RS, HGPS and WS hMSCs were infected at a low multiplicity of infection (MOI≈0.3) to make sure that one cell was infected with at most one sgRNA. 3 days after infection, puromycin (1 μg/mL) (InvivoGen) was added to select cells transduced with sgRNA. Then, hMSCs were continuously passaged to enrich

the sgRNAs that rejuvenate hMSCs to become dominant. With cell culturing for 36 days (RS hMSC) or 46 days (WS hMSC) or 54 days (HGPS hMSC), cells were harvested and the genomic DNA was extracted by DNeasy Blood & Tissue Kit (QIAGEN) following the manufacturer's protocol. Then the genomic DNA was sequenced, and the initial point was assessed by plasmid sequencing.

PCR was performed by PrimeSTAR HS DNA Polymerase (Takara) following the manufacturer's instructions. A two-step PCR process was used to amplify inserted sgRNA sequences, appended molecular barcodes and Illumina adapter sequences. The reaction conditions of the first PCR step: a) denaturation at 98°C, 2 min; b) 19 cycles at 98°C, 10 s; c) annealing at 58.0°C, 30 s; d) extension at 72°C, 40 s; e) final single extension step at 72°C, 5 min. After that, the amplicons were purified by C1 beads (Thermo Fisher Scientific) and then used as the template for the second PCR step. Illumina HiSeq 2500 adapter and barcode sequences were appended to the primers used in the second PCR step. The reaction conditions were the same as the first PCR step except for 14 cycles in step b). The primers are listed in Table S3.

Ultraviolet (UV)-, Oncogene-, and H_2O_2-induced cellular senescence

Cellular senescence induced by different cellular stresses was performed as previously described (Wang et al., 2021). For UV-induced cellular senescence, WT hMSCs were irradiated at 10 J/m^2 by XL-1000 UV Crosslinker (Spectronics Corporation). For oncogene-induced cellular senescence, WT hMSCs were transduced with pBABE-puro-Ras^{V12} retrovirus. For H_2O_2-induced cellular senescence, we treated WT hMSCs with 30 μM H_2O_2 for 24 h. When the cells reached 95% confluence, cells were passaged and processed for phenotypic analyses.

SA-β-gal staining

SA-β-gal staining was performed as previously described (Kubben et al., 2016). Cells were fixed with the fixation buffer (PBS with 2% formaldehyde and 0.2% glutaraldehyde) for 5 min at room temperature (RT). Then, cells were stained by fresh staining buffer containing X-gal at 37°C overnight. The percentage of positive cells was calculated and analyzed by ImageJ. Three biological replicates are performed for each group (n = 3).

Clonal expansion assay

Clonal expansion assay in hMSCs was performed as previously described (Li et al., 2020). 2,000 cells were seeded in a 12-well plate and cultured for about 2 weeks. Then, cells were fixed by 4% paraformaldehyde (PFA) for 30 min, followed by staining with 0.2% crystal violet for 1 hr at RT. Relative cell density

was quantified with ImageJ. Three biological replicates are performed for each group (*n* = 3).

Western blot analysis

Cells were lysed in SDS (Sigma-Aldrich) buffer as previously described (Bi et al., 2020), then a BCA kit (Thermo Fisher Scientific) was used to measure the protein concentration. 20 μg protein was subjected to SDS-PAGE and electrotransferred to PVDF membranes (Millipore). Then the membranes were blocked by 5% non-fat milk. After that, the membranes were incubated with specific primary antibodies overnight at 4°C, followed by HRP-conjugated secondary antibodies. The quantification of each image was detected by the ChemiDoc XRS+ system (Bio-Rad). GAPDH was used as a loading control. Three independent experiments were performed (*n* = 3) for each assay.

Antibodies used for western blot include anti-XPO7 (Santa Cruz Biotechnology, sc-390025), anti-HDAC2 (Abcam, ab16032), anti-GAPDH (Santa Cruz Biotechnology, sc-365062), anti-P21 (Cell Signaling Technology, 2947), anti-Lamin B1 (Abcam, ab16048), anti-LAP2 (BD Bioscience, 611000), anti-FLAG (Sigma, F1804), anti-P16 (BD Bioscience, 550834), HRP-conjugated goat anti-mouse IgG (ZSGB-BIO, ZB-2305), and HRP-conjugated goat anti-rabbit IgG (ZSGB-BIO, ZB-2301).

Immunofluorescence staining

Immunofluorescence staining was performed as previously described (Liu et al., 2012). Cells seeded on coverslips were fixed by 4% PFA for 30 min at RT, followed by washing with PBS and permeabilizing with 0.4% Triton X-100 (Sigma-Aldrich) for 10 min. Then 10% donkey serum was used to block cells for 1 hr at RT, and the indicated primary antibodies were incubated overnight at 4°C. After that, cells were washed by PBS and incubated with secondary antibodies and Hoechst33342 (Thermo Fisher Scientific) for 1 hr at RT. Images were taken by Zeiss LSM900 confocal microscope. For quantification of Ki67-positive cells, three biological replicates were set for each group (*n* = 3), and for quantification of H3K9me3 fluorescence intensity in each group was performed from 300 cells (hMSC) or 150 cells (primary fibroblast) from 3 biological replicates.

Antibodies used for immunofluorescence staining included anti-OCT4 (Santa Cruz Biotechnology, sc-5279), anti-SOX2 (R&D, MAB2018), anti-NANOG (Abcam, ab109250), anti-Ki67 (ZSGB-BIO, ZM-0166), anti-H3K9me3 (Abcam, ab8898), anti-γH2AX (Millipore, 05-636), Alexa 647 donkey anti-goat IgG (Thermo Fisher Scientific, A21447), Alexa 568 donkey anti-rabbit IgG (Thermo Fisher Scientific, A10042), and Alexa 488 donkey anti-mouse IgG (Thermo Fisher Scientific, A21202).

Cell cycle analysis

Cells were fixed with 70% ethanol overnight at -20°C. After that, cells were washed with PBS, permeabilized with 0.1% Triton X-100 and incubated with 0.2 mg/mL RNase A and stained with 0.02 mg/mL propidium iodide at 37°C for 30 min. The cell cycle analysis was conducted by BD LSRFortessa Cell Analyzer. Three biological replicates are performed for each group (n = 3).

Enzyme-Linked Immunosorbent Assay (ELISA)

The IL-6 secretion of hMSCs was detected by an ELISA kit (Biolegend, 430504) following the manufacturer's instructions. In brief, the medium was collected and incubated in an anti-IL6 antibody-coated plate. After that, Avidin-HRP, freshly mixed TMB substrate, and stop solution were added. Then the plate was measured at 450 nm. IL-6 levels were normalized to the corresponding cell numbers. Three biological replicates were performed for each group (n = 3)

RNA and DNA analyses

Total RNA was extracted by TRIzol™ (Thermo Fisher Scientific), after that GoScript™ Reverse Transcription System (Promega) was used to generate cDNA. After that, RT-qPCR was performed using the qPCR Mix (TOYOBO) in a CFX384 Real-Time system (Bio-Rad). *GAPDH* was used as an internal control. Four biological replicates were performed for each group (n = 4). DNeasy Blood & Tissue Kit (QIAGEN) was used to extract the genomic DNA, and the PCR amplification was performed by PrimeSTAR DNA Polymerase. The primers used for qPCR and PCR are listed in Table S3.

For RNA-seq, 1~2 µg of total RNA was quantified using the fragment analyzer (Advanced Analytical) and sequenced with Novaseq 6000.

Co-immunoprecipitation (Co-IP)

Co-IP assay was performed as previously reported (Diao et al., 2021). HEK293T cells were transfected with plasmids expressing Flag- XPO7 or Flag-Luc. Then cells were lysed in CHAPS lysis buffer (120 mM NaCl, 0.3% CHAPS, 40 mM HEPES (pH 7.5), 1 mM EDTA, and protease inhibitor cocktail (Roche)) at 4°C for 2 hr and centrifuged at 12,000 g, 4°C for 30 min. The supernatants were incubated with anti-FLAG Affinity Gel (Sigma, A2220) at 4°C overnight. FLAG peptides were used to obtain the XPO7-interacting protein complexes, and processed for western blot or LC-MS/MS analysis. For endogenous co-IP assays, after cell lysis, the supernatants were mixed with indicated antibodies and rotated at 4°C overnight. After that, the supernatants were incubated with beads at 4°C for 3 hr. Then, the immunocomplexes were washed three times with CHAPS lysis buffer and processed for western blot.

LC-MS/MS analysis

The eluted proteins obtained by Co-IP were subjected to 10% SDS-PAGE gel and stained by Coomassie brilliant blue. Then the gel bands were excised, cut into small plugs, dehydrated (100% acetonitrile), reduced (10 mM DTT in 25 mM NH_4HCO_3 at 56°C for 45 min) and alkylated (40 mM iodoacetamide in 25 mM NH_4HCO_3 at RT for 45 min in the dark). After that, the gel plugs were dried and digested with sequencing-grade modified trypsin (40 ng per band) in 25 mM NH_4HCO_3 at 37°C overnight. Formic acid was used to terminate the enzymatic reaction. The nanoLC-MS/MS experiments were performed on Q Exactive mass spectrometer (Thermo Scientific). Then, the raw data from Q Exactive analysis were analyzed with Proteome Discovery (2.2.0.388) using the Sequest HT search engine for protein identification. The data were searched against the UniProt human protein database (update-180920). False discovery rate (FDR) < 1% was set as the threshold for protein identification, and the peptide confidence was set to high for peptide filtering.
XPO7-interacting proteins are listed in Table S2.

CRISPR-based screening analysis

Sequencing data were analyzed with MAGeCK (version 0.5.9.2). Read counts were initially obtained from samples by "count" subcommand in MAGeCK, in which the data quality was accessed by fastqc, and reads with high-quality were further mapped to the screening library. Then, compared with the count of baseline library, the positively and negatively selected sgRNAs were identified by the "test" subcommand. Finally, sgRNAs with *P*-value < 0.05 were considered as significant and ranked by RRA (robust rank aggregation) score in positive selection.

RNA-seq analysis

For RNA-seq raw data, sequences with adaptors and low-quality reads were removed by Trim Galore (version 0.4.5). Clean data was mapped to the human hg19 reference genome by STAR (version 2.7.1a) software with default parameters (Dobin et al., 2013). The reads mapped to each gene were calculated using featureCounts (version 2.0.1) (Liao et al., 2014). Differentially expressed genes (DEGs) were calculated using the R package DESeq2 (version 1.30.1) with the cutoff of adjusted *P*-value less than 0.05 and $|\log_2$ (fold change)$|$ more than 0.5 (Love et al., 2014). Gene Ontology (GO) terms and pathways enrichment analysis was performed by Metascape (Zhou et al., 2019).

Copy number variation (CNV) analysis

For CNV data analysis, raw reads were trimmed with TrimGalore (version 0.4.5), then aligned to the human hg19 reference genome by Bowtie2 (version

2.4.2) (Langmead and Salzberg, 2012). The mapping reads are counted for each 500-kb bin using readCounter function of hmmcopy_utils (https://github.com/shahcompbio/hmmcopy_utils). The R/Bioconductor package HMMcopy (version 1.26.0) was used to correct copy number, GC content and mappability.

Statistical analysis

Statistical analyses were performed using Two-tailed Student's *t*-test with GraphPad Prism (version 8.0). Results are shown as the means ± SEM. *P*-value < 0.05 (*), *P*-value < 0.01 (**), and *P*-value < 0.001 (***) are considered as statistically significant.

Data Availability

The screening and transcriptomic data obtained in the study have been deposited in the Genome Sequence Archive in the National Genomics Data Center, Beijing Institute of Genomics (China National Center for Bioinformation) of the Chinese Academy of Sciences (Chen et al., 2021), under accession number HRA003304 and HRA003369. The LC-MS/MS data have been deposited to the ProteomeXchange Consortium (http://proteomecentral.proteomexchange.org) via the iProX partner repository (Ma et al., 2019) under accession number PXD037890.

Supplemental Figure Legends

Figure S1. CRISPR-based screening identifies XPO7 as a driver of cellular senescence.

(A) Table summarizing the quality of the NTSAG library.
(B) Bar plots showing the percentage of mapped reads by CRISPR-based screening in RS, WS and HGPS hMSCs.
(C) Bar plots showing the number of mapped reads per sgRNA by CRISPR-based screening in RS, WS and HGPS hMSCs.
(D) Heatmap showing the RT-qPCR detection of the relative mRNA levels for the indicated genes in RS hMSCs after CRISPR-mediated knockout (CRISPRko) of XPO7.
(E) ELISA analysis for the secretion of interleukin-6 (IL-6) in RS hMSCs after CRISPR-mediated knockout (CRISPRko) of XPO7. Data are presented as the mean ± SEM. *n* = 3 biological replicates. *, *P* < 0.05.
(F) Immunofluorescence analysis of H3K9me3 in RS hMSCs after CRISPR-mediated knockout (CRISPRko) of XPO7. Scale bars, 20 μm. The white arrowheads denote the cells with increased H3K9me3 signals. Data are presented as the mean ± SEM. *n* = 300 cells from three biological replicates. ***, *P* < 0.001.

(G) Immunofluorescence analysis of γ.H2AX foci in RS hMSCs after CRISPR-mediated knockout (CRISPRko) of XPO7. Scale bars, 20 μm. White arrows indicate γ.H2AX-positive cells. Data are presented as the mean ± SEM. n = 3 biological replicates. *, P < 0.05.
(H) ELISA analysis for the secretion of interleukin-6 (IL-6) in WS hMSCs after CRISPR-mediated knockout (CRISPRko) of XPO7. Data are presented as the mean ± SEM. n = 3 biological replicates. *, P < 0.05.
(I) ELISA analysis for the secretion of interleukin-6 (IL-6) in HGPS hMSCs after CRISPR-mediated knockout (CRISPRko) of XPO7. Data are presented as the mean ± SEM. n = 3 biological replicates. **, P < 0.01.

Figure S2. Generation and characterization of $XPO7^{-/-}$ hESCs and hMSCs.

(A) Schematic diagram of XPO7 knockout strategy through CRISPR/Cas9-mediated non-homologous end-joining (NHEJ) in $XPO7^{+/+}$ hESCs. Sequencing results showed a 1-bp (A/T) insertion introduced by genome editing.
(B) Western blot analysis of XPO7 in $XPO7^{+/+}$ and $XPO7^{-/-}$ hESCs.
(C) Immunofluorescence analysis of Ki67 in $XPO7^{+/+}$ and $XPO7^{-/-}$ hESCs. Scale bars, 20 μm. Data are presented as the means ± SEM. n = 3 biological replicates. ns, not significant.
(D) Immunofluorescence analysis of pluripotency markers OCT4, SOX2 and NANOG in $XPO7^{+/+}$ and $XPO7^{-/-}$ hESCs. Scale bars, 20 μm. Phase-contrast images were shown on the left. Scale bars, 250 μm.
(E) Copy number variation (CNV) analysis of $XPO7^{+/+}$ and $XPO7^{-/-}$ hESCs.
(F) Karyotype analysis of $XPO7^{-/-}$ hESCs.
(G) Schematic diagram showing hMSCs differentiation from corresponding hESCs.
(H) Flow cytometric analysis of CD73, CD90 and CD105 in $XPO7^{+/+}$ and $XPO7^{-/-}$ hMSCs.
(I) Copy number variation (CNV) analysis of $XPO7^{+/+}$ and $XPO7^{-/-}$ hMSCs.
(J) Analysis of the osteogenesis capacity of $XPO7^{+/+}$ and $XPO7^{-/-}$ hMSCs by von Kossa staining. Scale bars, 100 μm. Data are presented as the means ± SEM. n = 3 biological replicates. *, P < 0.05.
(K) Analysis of the adipogenesis capacity of $XPO7^{+/+}$ and $XPO7^{-/-}$ hMSCs by Oil Red O staining. Scale bars, 100 μm. Data are presented as the means ± SEM. n = 3 biological replicates. ns, not significant.
(L) Analysis of the chondrogenesis capacity of $XPO7^{+/+}$ and $XPO7^{-/-}$ hMSCs by Toluidine Blue staining. Scale bars, 100 μm. Data are presented as means ± SEM. n= 3 biological replicates. ns, not significant.
(M) ELISA analysis for the secretion of interleukin-6 (IL-6) in $XPO7^{+/+}$ and $XPO7^{-/-}$ hMSCs. Data are presented as the mean ± SEM. n = 3 biological replicates. ***, P < 0.001.

(N) Heatmap showing the Euclidian distance between replicates of RNA-seq *XPO7*$^{+/+}$ and *XPO7*$^{-/-}$ hMSCs. The color keys of the Euclidean distance from blue to white indicate strong to weak correlations.
(O) Volcano plot showing the upregulated (pink) and downregulated (green) differentially expressed genes (DEGs) in *XPO7*$^{-/-}$ hMSCs.
(P) Heatmap showing downregulated SASP-associated DEGs in *XPO7*$^{-/-}$ hMSCs. The color key from green to gray indicates z-score from low to high.

Figure S3. XPO7 regulates cellular senescence induced by different stressors.

(A) Schematic diagram of studies of the role of XPO7 in regulating cellular senescence in hMSCs induced by ultraviolet (UV) light.
(B) SA-β-gal staining of sg-*XPO7*-transduced hMSCs after UV irradiation. Scale bars, 100 μm. Data are presented as the mean ± SEM. n = 3 biological replicates. **, $P < 0.01$.
(C) Immunofluorescence analysis of Ki67 in sg-*XPO7*-transduced hMSCs after UV irradiation. Scale bars, 20 μm. White arrows indicate Ki67-positive cells. Data are presented as the means ± SEM. n = 3 biological replicates. ***, $P < 0.001$.
(D) Clonal expansion analysis of sg-*XPO7*-transduced hMSCs after UV irradiation. Data are presented as the means ± SEM. n = 3 biological replicates. *, $P < 0.05$; ***, $P < 0.001$.
(E) Schematic diagram of studies of the role of XPO7 in regulating cellular senescence in hMSCs upon H-Ras^{v12} overexpression.
(F) SA-β-gal staining of sg-*XPO7*-transduced hMSCs after H-Ras^{v12} overexpression. Scale bars, 100 μm. Data are presented as the mean ± SEM. n = 3 biological replicates. **, $P < 0.01$.
(G) Immunofluorescence analysis of Ki67 in sg-*XPO7*-transduced hMSCs after H-Ras^{v12} overexpression. Scale bars, 20 μm. White arrows indicate Ki67-positive cells. Data are presented as the means ± SEM. n = 3 biological replicates. *, $P < 0.05$; **, $P < 0.01$.
(H) Clonal expansion analysis of sg-*XPO7*-transduced hMSCs after H-Ras^{v12} overexpression. Data are presented as the means ± SEM. n = 3 biological replicates. *, $P < 0.05$; ***, $P < 0.001$.
(I) Schematic diagram of studies of the role of *XPO7* in regulating cellular senescence in hMSCs upon H_2O_2 treatment.
(J) SA-β-gal staining of sg-*XPO7*-transduced hMSCs after H_2O_2 treatment. Scale bars, 100 μm. Data are presented as the mean ± SEM. n = 3 biological replicates. **, $P < 0.01$.
(K) Immunofluorescence analysis of Ki67 in sg-*XPO7*-transduced hMSCs after H_2O_2 treatment. Scale bars, 20 μm. White arrows indicate Ki67-positive cells. Data are presented as the means ± SEM. n = 3 biological replicates. ***, $P < 0.001$.

(L) Clonal expansion analysis of sg-*XPO7*-transduced hMSCs after H_2O_2 treatment. Data are presented as the means ± SEM. *n* = 3 biological replicates. *, *P* < 0.05; **, *P* < 0.01.

Figure S4. XPO7 regulates human primary fibroblast senescence.

(A) SA-β-gal staining of human primary fibroblasts after CRISPR-mediated knockout (CRISPRko) of XPO7. Scale bars, 100 μm. Data are presented as the mean ± SEM. *n* = 3 biological replicates. **, *P* < 0.01.
(B) Immunofluorescence analysis of Ki67 in human primary fibroblasts after CRISPR-mediated knockout (CRISPRko) of XPO7. Scale bars, 20 μm. White arrows indicate Ki67-positive cells. Data are presented as the mean ± SEM. *n* = 3 biological replicates. *, *P* < 0.05.
(C) Clonal expansion analysis of human primary fibroblasts after CRISPR-mediated knockout (CRISPRko) of XPO7. Data are presented as the mean ± SEM. *n* = 3 biological replicates. ***, *P* < 0.001.
(D) Immunofluorescence analysis of H3K9me3 in human fibroblasts after CRISPR-mediated knockout (CRISPRko) of XPO7. Scale bars, 20 μm. White arrowheads denote the cells with increased H3K9me3 signals. Data are presented as the mean ± SEM. *n* =150 cells from three biological replicates. ***, *P* < 0.001.
(E) SA-β-gal staining of human primary fibroblasts after CRISPRa-mediated transcriptional activation of XPO7. Scale bars, 100 μm. Data are presented as the mean ± SEM. *n* = 3 biological replicates. **, *P* < 0.01.
(F) Immunofluorescence analysis of Ki67 in human primary fibroblasts after CRISPRa-mediated transcriptional activation of XPO7. Scale bars, 20 μm. White arrows indicate Ki67-positive cells. Data are presented as the mean ± SEM. *n* = 3 biological replicates. **, *P* < 0.01.
(G) Clonal expansion analysis of human primary fibroblasts after CRISPRa-mediated transcriptional activation of XPO7. Data are presented as the mean ± SEM. *n* = 3 biological replicates. **, *P* < 0.01.
(H) Immunofluorescence analysis of H3K9me3 in human primary fibroblasts after CRISPRa-mediated transcriptional activation of XPO7. Scale bars, 20 μm. White arrowheads denote the cells with increased H3K9me3 signals. Data are presented as the mean ± SEM. *n* = 150 cells from three biological replicates. ***, *P* < 0.001.

Figure S5. XPO7 promotes cellular senescence in early-passage hMSCs.

(A) RT-qPCR analysis of *XPO7* in young hMSCs after CRISPR activation (CRISPRa)-mediated transcriptional activation. Data are presented as the mean ± SEM. *n* = 4 biological replicates. **, *P* < 0.01.

(B) Western blot analysis of XPO7 in young hMSCs after CRISPR activation (CRISPRa)-mediated transcriptional activation. Data are presented as the mean ± SEM. *n* = 3 independent experiments. ***, *P* < 0.001.
(C) SA-β-gal staining of young hMSCs after CRISPRa-mediated transcriptional activation of XPO7. Scale bars, 100 μm. Data are presented as the mean ± SEM. *n* = 3 biological replicates. *, *P* < 0.05.
(D) Immunofluorescence analysis of Ki67 in young hMSCs after CRISPRa-mediated transcriptional activation of XPO7. Scale bars, 20 μm. White arrows indicate Ki67-positive cells. Data are presented as the mean ± SEM. *n* = 3 biological replicates. *, *P* < 0.05.
(E) Clonal expansion analysis of young hMSCs after CRISPRa-mediated transcriptional activation of XPO7. Data are presented as the mean ± SEM. *n* = 3 biological replicates. *, *P* < 0.05.
(F) Heatmap showing the RT-qPCR detection of the relative mRNA levels for the indicated genes in young hMSCs after CRISPRa-mediated transcriptional activation of XPO7.
(G) Western blot analysis of the indicated protein levels in young hMSCs after CRISPRa-mediated transcriptional activation of XPO7. Data are presented as the mean ± SEM. *n* = 3 independent experiments. *, *P* < 0.05.
(H) ELISA analysis for the secretion of interleukin-6 (IL-6) in young hMSCs after CRISPRa-mediated transcriptional activation of XPO7. Data are presented as the mean ± SEM. *n* = 3 biological replicates. *, *P* < 0.05.
(I) Immunofluorescence analysis of H3K9me3 in young hMSCs after CRISPRa-mediated transcriptional activation of XPO7. Scale bars, 20 μm. White arrowheads denote the cells with increased H3K9me3 signals. Data are presented as the mean ± SEM. *n* = 300 cells from three biological replicates. ***, *P* < 0.001.
(J) Western blot analysis of XPO7 in young hMSCs transduced with lentiviruses expressing Luc or XPO7. Data are presented as the mean ± SEM. *n* = 3 independent experiments. ***, *P* < 0.001.
(K) SA-β-gal staining of young hMSCs transduced with lentiviruses expressing Luc or XPO7. Scale bars, 100 μm. Data are presented as the mean ± SEM. *n* = 3 biological replicates. **, *P* < 0.01.
(L) Clonal expansion analysis of young hMSCs transduced with lentiviruses expressing Luc or XPO7. Data are presented as the mean ± SEM. *n* = 3 biological replicates. *, *P* < 0.05.
(M) Heatmap showing the RT-qPCR detection of the relative mRNA levels for the indicated genes in young hMSCs transduced with lentiviruses expressing Luc or XPO7.
(N) Western blot analysis of the indicated protein levels in young hMSCs transduced with lentiviruses expressing Luc or XPO7. Data are presented as the mean ± SEM. *n* = 3 independent experiments. *, *P* < 0.05; **, *P* < 0.01.

Figure S6. XPO7 regulates HDAC2 at the protein level.

(A) Scatter plot showing the expression levels (fragments per kilobase per million, FPKM) of the *HDAC2* gene in human fibroblasts from 133 individuals aged 1 to 94 years old. The linear regression line (with a 95% confidence interval) for *HDAC2* gene expression is colored in green (with light green shading). Pearson's correlation coefficient (R) and P value are shown as indicated. These data were obtained from a previous study (Fleischer et al., 2018). The gender-adjusted linear regression coefficient for age was -0.23, and the *P*-value was 0.013.
(B) RT-qPCR analysis of *HDAC2* in $XPO7^{+/+}$ and $XPO7^{-/-}$ hMSCs. Data are presented as the mean ± SEM. *n* = 4 biological replicates. ns, not significant.
(C) RT-qPCR analysis of *HDAC2* in RS hMSCs after CRISPR-mediated knockout (CRISPRko) of XPO7. Data are presented as the mean ± SEM. *n* = 4 biological replicates. ns, not significant.
(D) Western blot analysis of HDAC2 in RS hMSCs after CRISPR-mediated knockout (CRISPRko) of XPO7. Data are presented as the mean ± SEM. *n* = 3 independent experiments. **, *P* < 0.01.
(E) RT-qPCR analysis of *HDAC2* in WS hMSCs after CRISPR-mediated knockout (CRISPRko) of XPO7. Data are presented as the mean ± SEM. *n* = 4 biological replicates. ns, not significant.
(F) Western blot analysis of HDAC2 in WS hMSCs after CRISPR-mediated knockout (CRISPRko) of XPO7. Data are presented as the mean ± SEM. *n* = 3 independent experiments. *, *P* < 0.05.
(G) RT-qPCR analysis of *HDAC2* in HGPS hMSCs after CRISPR-mediated knockout (CRISPRko) of XPO7. Data are presented as the mean ± SEM. *n* = 4 biological replicates. ns, not significant.
(H) Western blot analysis of HDAC2 in HGPS hMSCs after CRISPR-mediated knockout (CRISPRko) of XPO7. Data are presented as the mean ± SEM. *n* = 3 independent experiments. *, *P* < 0.05.
(I) Western blot analysis of HDAC2 in young hMSCs after CRISPRa-mediated transcriptional activation of XPO7. Data are presented as the mean ± SEM. *n* = 3 independent experiments. **, *P* < 0.01.
(J) Protein stability analysis of HDAC2 after overexpression of XPO7. Protein levels of HDAC2 at indicated time points after treatment with 20 μg/mL cycloheximide (CHX) were determined by western blotting. Data are presented as the mean ± SEM. *n* = 3 independent replicates. *, *P* < 0.05.
(K) Western blot analysis of HDAC2 in WT hMSCs expressing Luc or XPO7 with or without the treatment of MG132 (10 μM). Data are presented as the mean ± SEM. *n* = 3 independent replicates. **, *P* < 0.01; ***, *P* < 0.001.
(L) Western blot analysis of HDAC2 protein level in hMSCs after CRISPR-mediated knockout (CRISPRko) of HDAC2. Data are presented as the mean ± SEM. *n* = 3 independent experiments. ***, *P* < 0.001.
(M) SA-β-gal staining of hMSCs after CRISPR-mediated knockout (CRISPRko) of HDAC2. Scale bars, 100 μm. Data are presented as the mean ± SEM. *n* = 3 biological replicates. ***, *P* < 0.001.

(N) Clonal expansion analysis of hMSCs after CRISPR-mediated knockout (CRISPRko) of HDAC2. Data are presented as the mean ± SEM. n = 3 biological replicates. **, $P < 0.01$.
(O) Immunofluorescence analysis of Ki67 in hMSCs after CRISPR-mediated knockout (CRISPRko) of HDAC2. Scale bars, 20 μm. White arrows indicate Ki67-positive cells. Data are presented as the mean ± SEM. n = 3 biological replicates. **, $P < 0.01$.
(P) Western blot analysis of the indicated protein levels in hMSCs after CRISPR-mediated knockout (CRISPRko) of HDAC2. Data are presented as the mean ± SEM. n = 3 independent experiments. **, $P < 0.01$; ***, $P < 0.001$.

Supplementary Table Legends

Table S1. Gene rank of CRISPR/Cas9-based LOF screening and differentially expressed genes (DEGs) in $XPO7^{-/-}$ hMSCs.
Table S2. Candidate XPO7-interacting proteins identified by LC-MS/MS.
Table S3. Sequence information of sgRNAs and primers used for CRISPR/Cas9-based screen library construction, gene editing, plasmid construction, RT-qPCR analysis as well as the donor information of human primary fibroblast.

References

Bi, S., Liu, Z., Wu, Z., Wang, Z., Liu, X., Wang, S., Ren, J., Yao, Y., Zhang, W., Song, M., *et al.* (2020). SIRT7 antagonizes human stem cell aging as a heterochromatin stabilizer. Protein Cell *11*, 483-504.

Chen, T., Chen, X., Zhang, S., Zhu, J., Tang, B., Wang, A., Dong, L., Zhang, Z., Yu, C., Sun, Y., *et al.* (2021). The Genome Sequence Archive Family: Toward Explosive Data Growth and Diverse Data Types. Genomics Proteomics Bioinformatics *19*, 578-583.

Diao, Z., Ji, Q., Wu, Z., Zhang, W., Cai, Y., Wang, Z., Hu, J., Liu, Z., Wang, Q., Bi, S., *et al.* (2021). SIRT3 consolidates heterochromatin and counteracts senescence. Nucleic Acids Res *49*, 4203-4219.

Dobin, A., Davis, C.A., Schlesinger, F., Drenkow, J., Zaleski, C., Jha, S., Batut, P., Chaisson,

M., and Gingeras, T.R. (2013). STAR: ultrafast universal RNA-seq aligner. Bioinformatics *29*, 15-21.

Fleischer, J.G., Schulte, R., Tsai, H.H., Tyagi, S., Ibarra, A., Shokhirev, M.N., Huang, L., Hetzer, M.W., and Navlakha, S. (2018). Predicting age from the transcriptome of human dermal fibroblasts. Genome Biol *19*, 221.

Kubben, N., Zhang, W., Wang, L., Voss, T.C., Yang, J., Qu, J., Liu, G.H., and Misteli, T. (2016). Repression of the Antioxidant NRF2 Pathway in Premature Aging. Cell *165*, 1361-1374.

Langmead, B., and Salzberg, S.L. (2012). Fast gapped-read alignment with Bowtie 2. Nat Methods *9*, 357-359.

Li, H., Wu, Z., Liu, X., Zhang, S., Ji, Q., Jiang, X., Liu, Z., Wang, S., Qu, J., Zhang, W.*, et al.* (2020). ALKBH1 deficiency leads to loss of homeostasis in human diploid somatic cells. Protein Cell *11*, 688-695.

Liang, C., Ke, Q., Liu, Z., Ren, J., Zhang, W., Hu, J., Wang, Z., Chen, H., Xia, K., Lai, X.*, et al.* (2022). BMAL1 moonlighting as a gatekeeper for LINE1 repression and cellular senescence in primates. Nucleic Acids Res *50*, 3323-3347.

Liao, Y., Smyth, G.K., and Shi, W. (2014). featureCounts: an efficient general purpose program for assigning sequence reads to genomic features. Bioinformatics *30*, 923-930.

Liu, G.H., Qu, J., Suzuki, K., Nivet, E., Li, M., Montserrat, N., Yi, F., Xu, X., Ruiz, S., Zhang, W.*, et al.* (2012). Progressive degeneration of human neural stem cells caused by pathogenic LRRK2. Nature *491*, 603-607.

Love, M.I., Huber, W., and Anders, S. (2014). Moderated estimation of fold change and dispersion for RNA-seq data with DESeq2. Genome Biol *15*, 550.

Ma, J., Chen, T., Wu, S., Yang, C., Bai, M., Shu, K., Li, K., Zhang, G., Jin, Z., He, F., *et al.* (2019). iProX: an integrated proteome resource. Nucleic Acids Res *47*, D1211-D1217.

Wang, W., Zheng, Y., Sun, S., Li, W., Song, M., Ji, Q., Wu, Z., Liu, Z., Fan, Y., Liu, F., *et al.* (2021). A genome-wide CRISPR-based screen identifies KAT7 as a driver of cellular senescence. Sci Transl Med *13*.

Wu, Z., Zhang, W., Song, M., Wang, W., Wei, G., Li, W., Lei, J., Huang, Y., Sang, Y., Chan, P., *et al.* (2018). Differential stem cell aging kinetics in Hutchinson-Gilford progeria syndrome and Werner syndrome. Protein Cell *9*, 333-350.

Zhang, S., Wu, Z., Shi, Y., Wang, S., Ren, J., Yu, Z., Huang, D., Yan, K., He, Y., Liu, X., *et al.* (2022). FTO stabilizes MIS12 and counteracts senescence. Protein Cell *13*, 954-960.

Zhang, W., Li, J., Suzuki, K., Qu, J., Wang, P., Zhou, J., Liu, X., Ren, R., Xu, X., Ocampo, A., *et al.* (2015). Aging stem cells. A Werner syndrome stem cell model unveils heterochromatin alterations as a driver of human aging. Science *348*, 1160-1163.

Zhou, Y., Zhou, B., Pache, L., Chang, M., Khodabakhshi, A.H., Tanaseichuk, O., Benner, C., and Chanda, S.K. (2019). Metascape provides a biologist-oriented resource for the analysis of systems-level datasets. Nat Commun *10*, 1523.

Zou, Z., Long, X., Zhao, Q., Zheng, Y., Song, M., Ma, S., Jing, Y., Wang, S., He, Y., Esteban, C.R., *et al.* (2021). A Single-Cell Transcriptomic Atlas of Human Skin Aging. Dev Cell *56*, 383-397 e388.

Figure S1

A

Quality control of NTSAG library

Number of perfect guide matches	12,052,254
Number of nonperfect guide matches	2,408,449
Number of reads where key was not found	326,390
Number of reads processed	14,787,093
Percentage of guides that matched perfectly	83.3
Percentage of undetected guides	0.0
Skew ratio of top 10% to bottom 10%	2.7

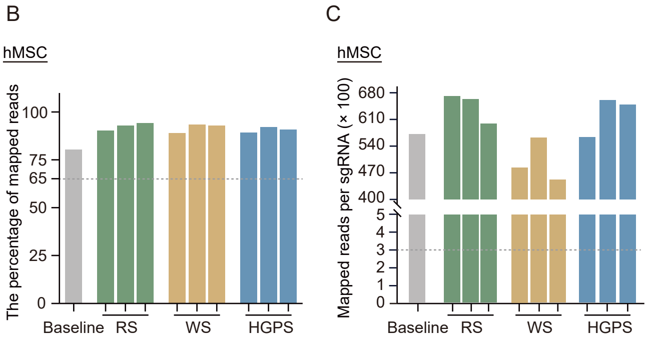

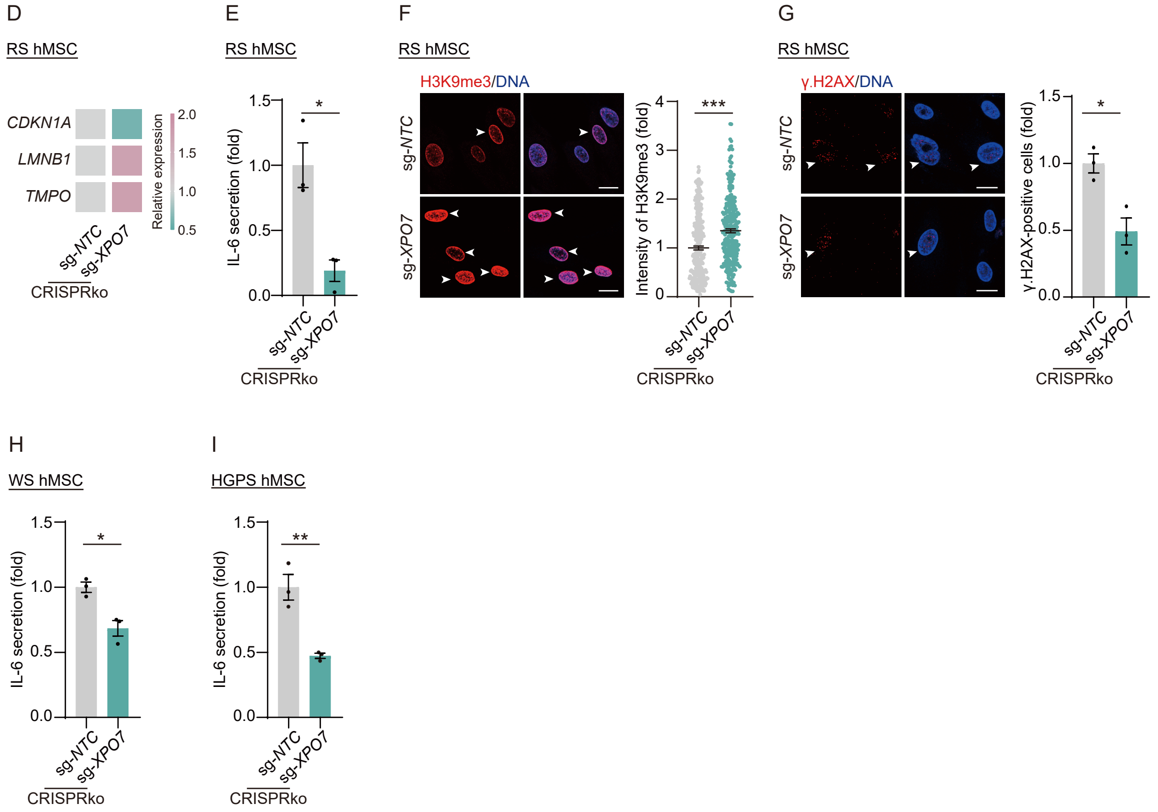

Figure S2

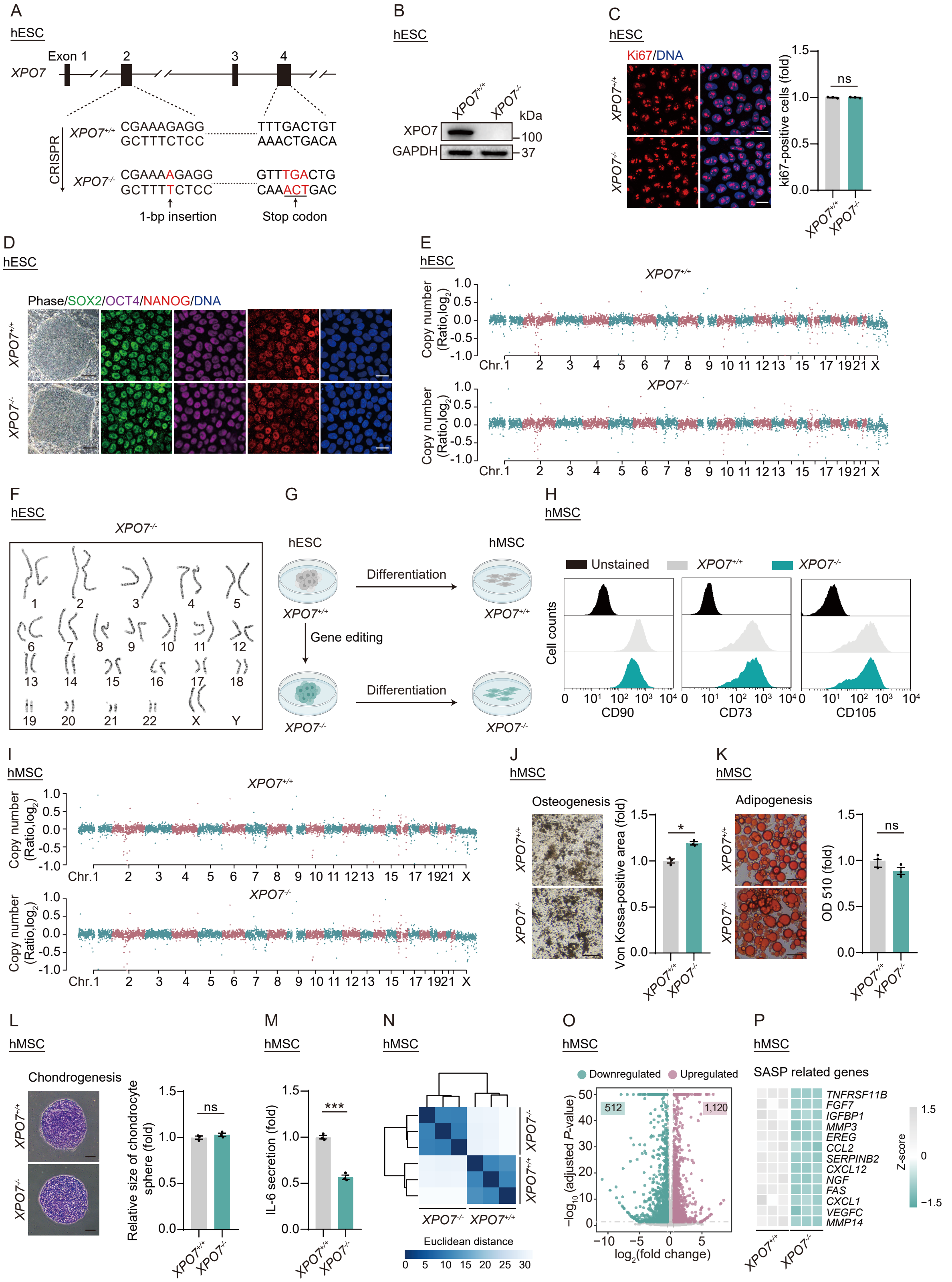
A
hESC
Exon 1
2
3
4
XPO7
CRISPR
XPO7+/+
CGAAAGAGG
GCTTTCTCC
TTTGACTGT
AAACTGACA
XPO7-/-
CGAAAAGAGG
GCTTTTCTCC
GTTTGACTG
CAAACTGAC
1-bp insertion
Stop codon
B
hESC
XPO7+/+
XPO7-/-
kDa
XPO7
GAPDH
100
37
C
hESC
Ki67/DNA
ki67-positive cells (fold)
ns
D
hESC
Phase/SOX2/OCT4/NANOG/DNA
E
hESC
Copy number (Ratio,log2)
Chr.
F
hESC
G
hESC
hMSC
Differentiation
Gene editing
H
hMSC
Unstained
Cell counts
CD90
CD73
CD105
I
hMSC
J
hMSC
Osteogenesis
Von Kossa-positive area (fold)
K
hMSC
Adipogenesis
OD 510 (fold)
L
hMSC
Chondrogenesis
Relative size of chondrocyte sphere (fold)
M
hMSC
IL-6 secretion (fold)
***
N
hMSC
Euclidean distance
O
hMSC
Downregulated
Upregulated
512
1,120
-log10 (adjusted P-value)
log2(fold change)
P
hMSC
SASP related genes
TNFRSF11B
FGF7
IGFBP1
MMP3
EREG
CCL2
SERPINB2
CXCL12
NGF
FAS
CXCL1
VEGFC
MMP14
Z-score

Figure S3

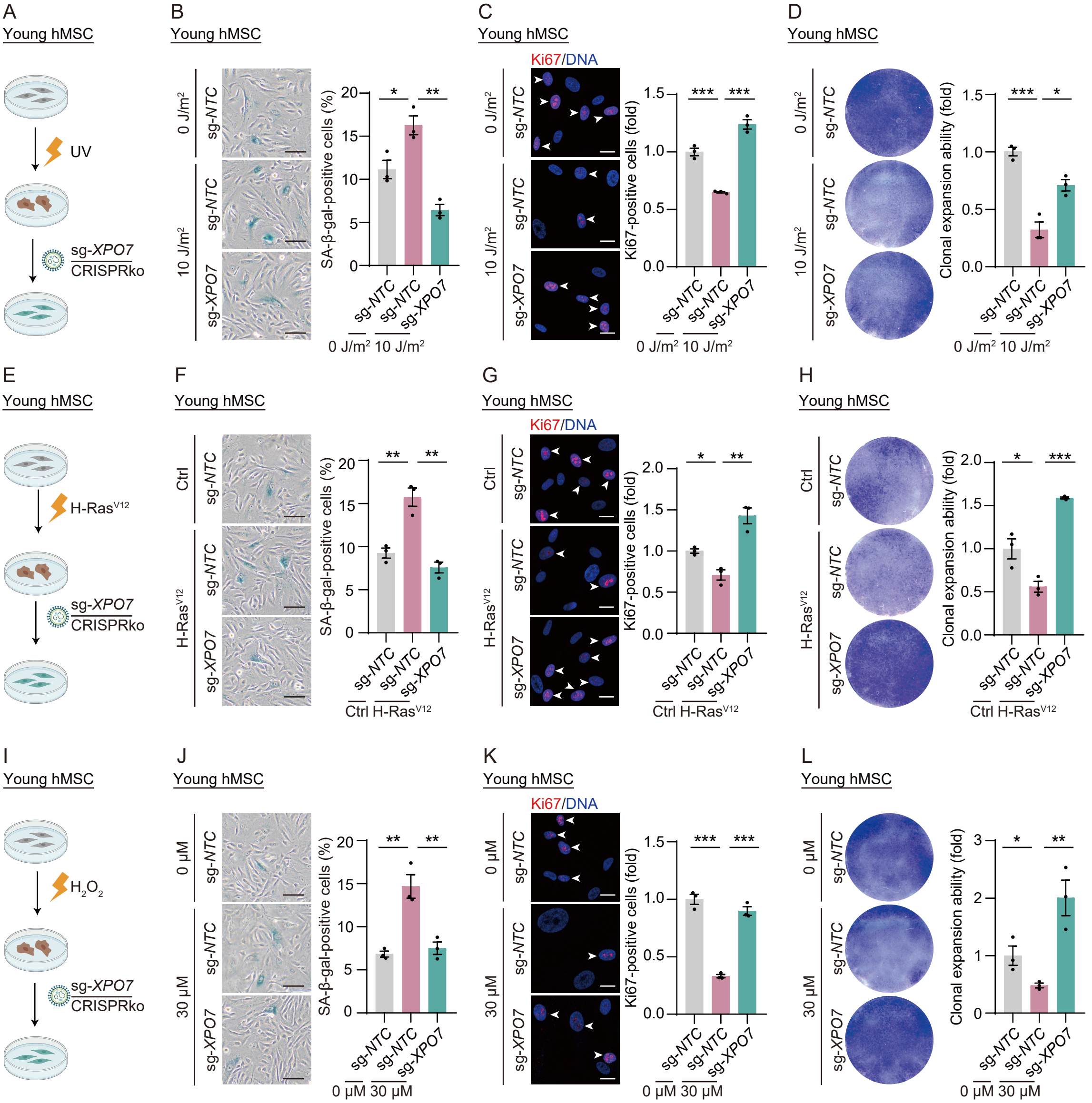
A
Young hMSC
UV
sg-XPO7
CRISPRko
B
Young hMSC
0 J/m²
10 J/m²
sg-NTC
sg-NTC
sg-XPO7
SA-β-gal-positive cells (%)
C
Young hMSC
Ki67/DNA
Ki67-positive cells (fold)
D
Young hMSC
Clonal expansion ability (fold)
E
Young hMSC
H-RasV12
F
Young hMSC
Ctrl
H-RasV12
G
Young hMSC
H
Young hMSC
I
Young hMSC
H2O2
J
Young hMSC
0 μM
30 μM
K
Young hMSC
L
Young hMSC

Figure S4

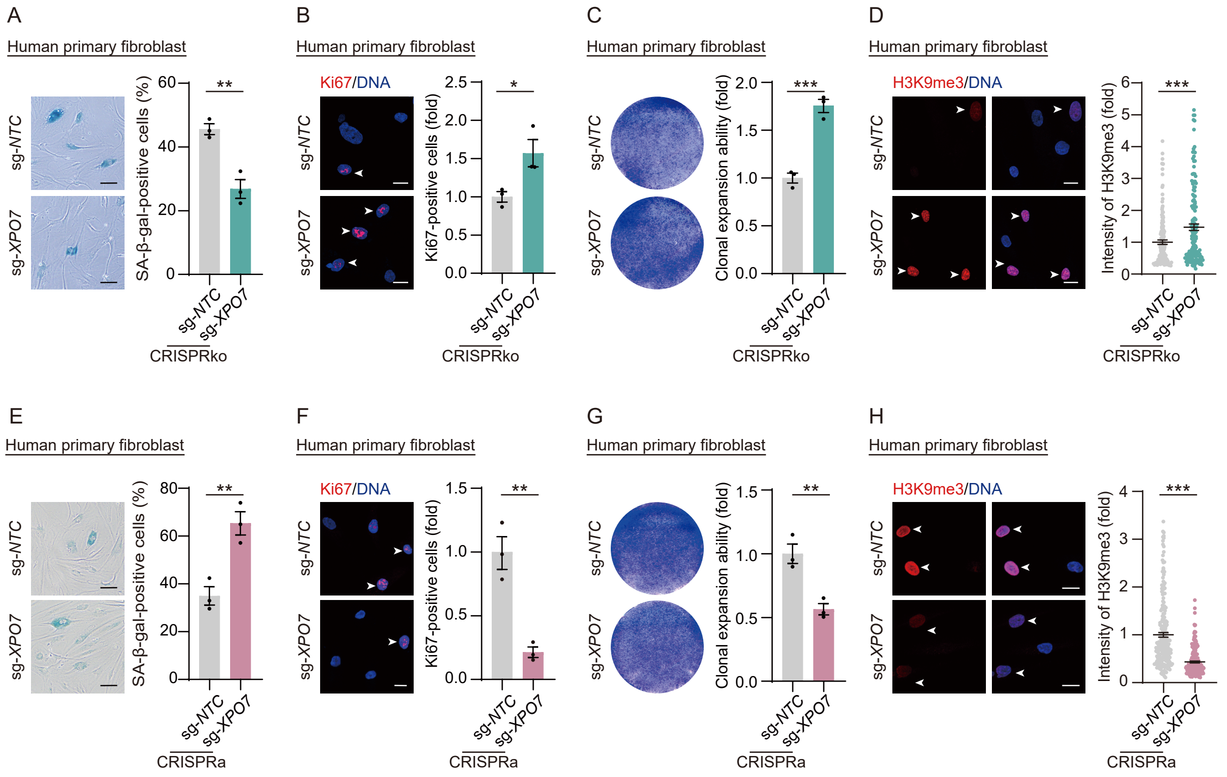
A
Human primary fibroblast
sg-NTC
sg-XPO7
SA-β-gal-positive cells (%)
0
20
40
60
**
CRISPRko
B
Human primary fibroblast
Ki67/DNA
Ki67-positive cells (fold)
0.0
0.5
1.0
1.5
2.0
2.5
*
CRISPRko
C
Human primary fibroblast
Clonal expansion ability (fold)
0.0
0.5
1.0
1.5
2.0
***
CRISPRko
D
Human primary fibroblast
H3K9me3/DNA
Intensity of H3K9me3 (fold)
0
1
2
3
4
5
6
***
CRISPRko
E
Human primary fibroblast
SA-β-gal-positive cells (%)
0
20
40
60
80
**
CRISPRa
F
Human primary fibroblast
Ki67/DNA
Ki67-positive cells (fold)
0.0
0.5
1.0
1.5
**
CRISPRa
G
Human primary fibroblast
Clonal expansion ability (fold)
0.0
0.5
1.0
1.5
**
CRISPRa
H
Human primary fibroblast
H3K9me3/DNA
Intensity of H3K9me3 (fold)
0
1
2
3
4
***
CRISPRa

Figure S5

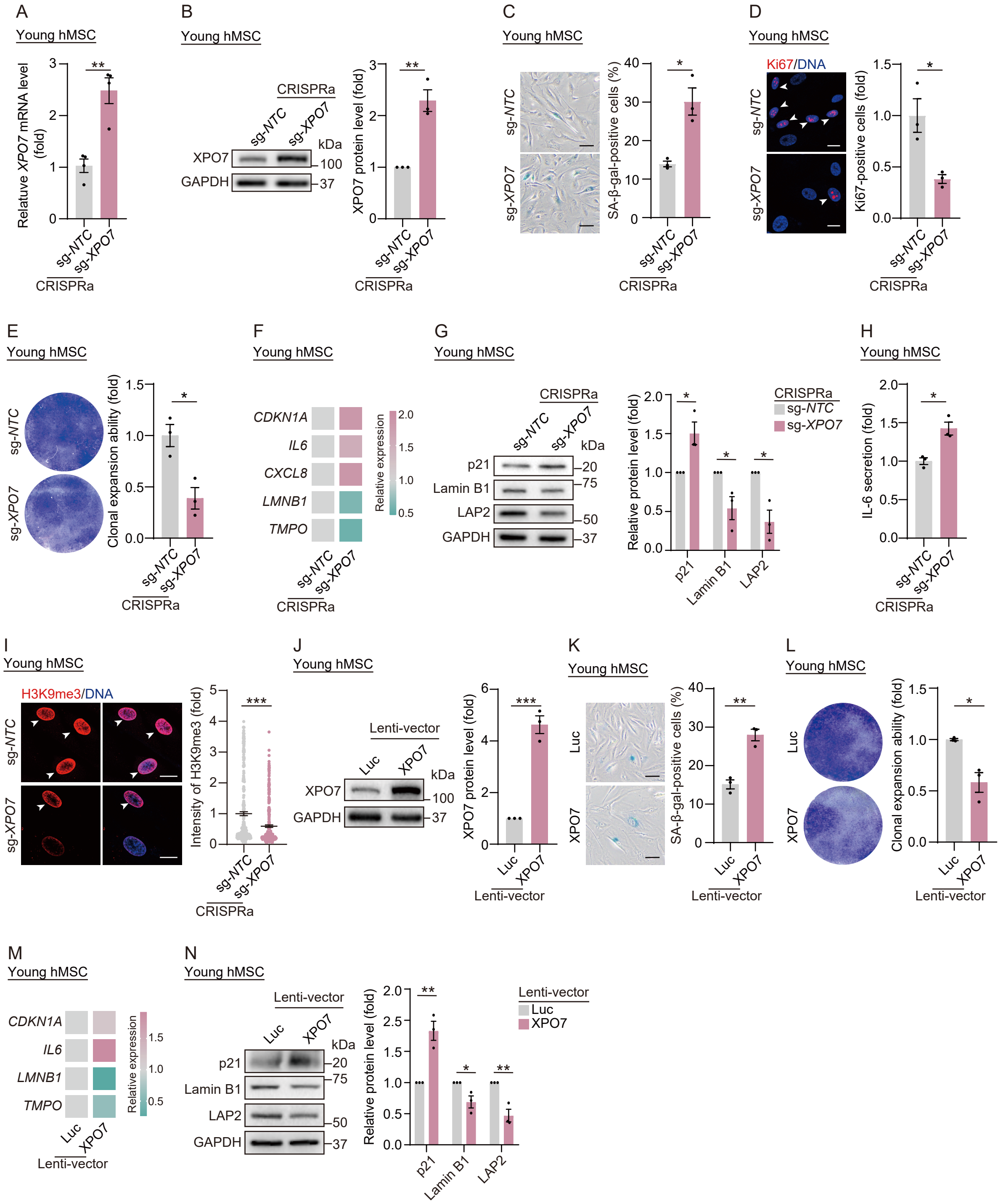
A
Young hMSC
Relature XPO7 mRNA level (fold)
**
sg-NTC
sg-XPO7
CRISPRa
B
Young hMSC
CRISPRa
sg-NTC
sg-XPO7
kDa
XPO7
100
GAPDH
37
XPO7 protein level (fold)
**
C
Young hMSC
sg-NTC
sg-XPO7
SA-β-gal-positive cells (%)
*
D
Young hMSC
Ki67/DNA
Ki67-positive cells (fold)
E
Young hMSC
Clonal expansion ability (fold)
F
Young hMSC
CDKN1A
IL6
CXCL8
LMNB1
TMPO
Relative expression
G
Young hMSC
p21
Lamin B1
LAP2
GAPDH
20
75
50
37
Relative protein level (fold)
sg-NTC
sg-XPO7
H
Young hMSC
IL-6 secretion (fold)
I
Young hMSC
H3K9me3/DNA
Intensity of H3K9me3 (fold)
***
J
Young hMSC
Lenti-vector
Luc
XPO7
XPO7 protein level (fold)
K
Young hMSC
Luc
XPO7
SA-β-gal-positive cells (%)
Lenti-vector
L
Young hMSC
Luc
XPO7
Clonal expansion ability (fold)
Lenti-vector
M
Young hMSC
CDKN1A
IL6
LMNB1
TMPO
Relative expression
Luc
XPO7
Lenti-vector
N
Young hMSC
Lenti-vector
Luc
XPO7
kDa
p21
Lamin B1
LAP2
GAPDH
Relative protein level (fold)
Lenti-vector
Luc
XPO7

Figure S6

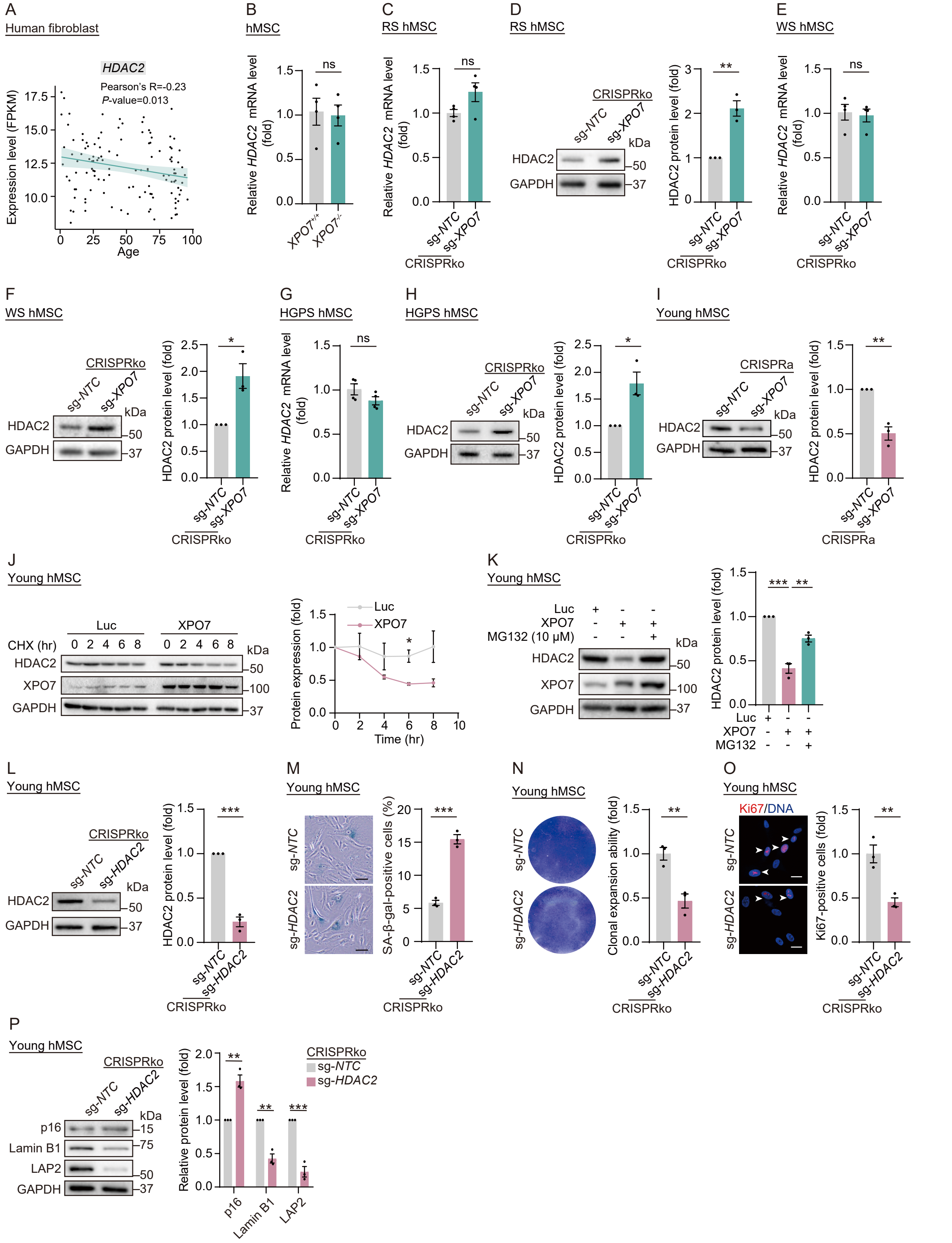

A
Human fibroblast
HDAC2
Pearson's R=-0.23
P-value=0.013
Expression level (FPKM)
Age
B
hMSC
Relative HDAC2 mRNA level (fold)
ns
XPO7+/+
XPO7-/-
C
RS hMSC
Relative HDAC2 mRNA level (fold)
ns
sg-NTC
sg-XPO7
CRISPRko
D
RS hMSC
CRISPRko
sg-NTC
sg-XPO7
kDa
HDAC2
GAPDH
HDAC2 protein level (fold)
**
CRISPRko
E
WS hMSC
Relative HDAC2 mRNA level (fold)
ns
CRISPRko
F
WS hMSC
CRISPRko
HDAC2
GAPDH
HDAC2 protein level (fold)
*
G
HGPS hMSC
Relative HDAC2 mRNA level (fold)
ns
H
HGPS hMSC
CRISPRko
HDAC2
GAPDH
HDAC2 protein level (fold)
*
I
Young hMSC
CRISPRa
HDAC2
GAPDH
HDAC2 protein level (fold)
**
J
Young hMSC
Luc
XPO7
CHX (hr)
HDAC2
XPO7
GAPDH
Protein expression (fold)
Time (hr)
K
Young hMSC
Luc
XPO7
MG132 (10 μM)
HDAC2
XPO7
GAPDH
HDAC2 protein level (fold)
***
**
L
Young hMSC
CRISPRko
sg-NTC
sg-HDAC2
HDAC2
GAPDH
HDAC2 protein level (fold)
***
M
Young hMSC
SA-β-gal-positive cells (%)
***
N
Young hMSC
Clonal expansion ability (fold)
**
O
Young hMSC
Ki67/DNA
Ki67-positive cells (fold)
**
P
Young hMSC
CRISPRko
p16
Lamin B1
LAP2
GAPDH
Relative protein level (fold)
sg-NTC
sg-HDAC2
